# Coping and compromise: a qualitative study of how primary health care providers respond to health reform in China

**DOI:** 10.1186/s12960-017-0226-z

**Published:** 2017-08-04

**Authors:** Mingji Zhang, Wei Wang, Ross Millar, Guohong Li, Fei Yan

**Affiliations:** 1 School of Public Health, Shanghai Jiaotong University, No. 227 South Chonqing Rd. Huangpu District, Shanghai, 200025 China; 2 Center for Health Technology Assessment, China Hospital Development Institute of Shanghai Jiaotong University, No. 227 South Chonqing Rd. Huangpu District, Shanghai, 200025 China; 3 School of Public Health, Fudan University, Shanghai, China; 4 Health Services Management Centre, University of Birmingham, Birmingham, United Kingdom

**Keywords:** Community health services, Primary health care, Work attitude, Coping strategy, Health reform

## Abstract

**Background:**

Health reform in China since 2009 has emphasized basic public health services to enhance the function of Community Health Services as a primary health care facility. A variety of studies have documented these efforts, and the challenges these have faced, yet up to now the experience of primary health care (PHC) providers in terms of how they have coped with these changes remains underdeveloped. Despite the abundant literature on psychological coping processes and mechanisms, the application of coping research within the context of human resources for health remains yet to be explored. This research aims to understand how PHC providers coped with the new primary health care model and the job characteristics brought about by these changes.

**Methods:**

Semi-structured interviews with primary health care workers were conducted in Jinan city of Shandong province in China. A maximum variation sampling method selected 30 PHC providers from different specialties. Thematic analysis was used drawing on a synthesis of theories related to the Job Demands-Resources model, work adjustment, and the model of exit, voice, loyalty and neglect to understand PHC providers’ coping strategies.

**Results:**

Our interviews identified that the new model of primary health care significantly affected the nature of primary health work and triggered a range of PHC providers’ coping processes. The results found that health workers perceived their job as less intensive than hospital medical work but often more trivial, characterized by heavy workload, blurred job description, unsatisfactory income, and a lack of professional development. However, close relationship with community and low work pressure were satisfactory. PHC providers’ processing of job demands and resources displayed two ways of interaction: aggravation and alleviation. Processing of job demands and resources led to three coping strategies: exit, passive loyalty, and compromise with new roles and functions.

**Conclusions:**

Primary health care providers employed coping strategies of exit, passive loyalty, and compromise to deal with changes in primary health work. In light of these findings, our paper concludes that it is necessary for the policymakers to provide further job resources for CHS, and involve health workers in policy-making. The introduction of particular professional training opportunities to support job role orientation for PHC providers is advocated.

## Background

### CHS development in China

Community health services (CHS) in China are facilities that provide primary health care to community residents within a particular locality. They were initiated in 1997 in a series of pilot sites [1] and carried out functions of preventive health services, medical services, health management, rehabilitation, health education, and family planning. Primary health care (PHC) providers were health workers in CHS. They were public employees, yet their financial input from government was scarce [2]. As a result, these providers were required to generate additional income from medical services through fee-for-service charges to make up cost of public health work that was provided free to residents [3]. Driven by financial motives, medical services subsequently became a significant part in CHS work, with much of CHS work mirroring that provided by first-level hospitals [4].

Since 2009, the keynote of China’s health reform has been to rebuild the healthcare system with CHS acting as gatekeepers providing primary health care [5, 6]. Rather than visiting any health care provider at will, patients are encouraged to first visit general practitioners (GPs) in the CHS [5, 7] with decisions then made about whether or not to refer on for further hospital care.

A new model of primary health care has started to take effect with the government advocating the development of a network of primary health facilities. These has had wide ranging implications for grass-root health care facilities, when many were transited from clinics in public enterprises, first- or second-level public hospitals, or indeed private clinics into the CHS. Thus, a diverse group of CHS came into existence, with various types of ownership, such as CHS owned by public enterprises, public hospitals, private owners, and the local health bureau [8].

In this health reform, the functions of CHS have been condensed into basic public health services (BPHS) and basic medical services (BMS) [9]. BPHS is funded from central and local governments [10] and has seen its role and remit increased. National guideline in 2009 stipulated 10 tasks of BPHS which included health archive management, health education, health management for children under 3, maternity services, elderly care services, vaccinations, communicable disease reporting, health management for diabetics and hypertension patients, and health management for severe psychotics [11]. It increased to include health management for children under 6, public health emergency reporting and assistance for health inspection in 2011, traditional Chinese medicine (TCM) in 2013, and services for tuberculosis patients in 2017 [12]. On the other hand, BMS—a traditional function for CHS provided by GPs and specialists—has seen no additional guidelines to clarify the range and standard of medical services at a primary level. There has been no national funding for BMS. Indeed, the launch of essential medicine programme narrowed the drugs that could be prescribed by these primary health facilities.

Within the context of these changes to CHS functions, PHC providers have undergone a role transformation. Before 2009, staff worked in a similar way to hospitals where specialists were assisted by nurses. After 2009, patterns changed with specialists now trained and transformed to GPs and new generations of general practitioners graduating from medical colleges [13]. Public health workers received specific training about BPHS, and work alongside GPs, nurses, and other cadres like TCM doctors. Only a few of public health workers were officially registered public health doctors, most were transited from nurses or clinical doctors after having accepted adequate training. The team is led by a GP and practices in primary health facilities, community public centers, and sometimes in residents’ homes. Besides teaming with public health workers, additional duties for GPs also includes BPHS follow-up and form-filling work during a patient’s visit [14]. In short, PHC provider, the majority of which appeared after 2009, was an extremely new profession among health workers in China.

These role changes have coincided with public finance rise for primary health services. Basically, PHC provider salary is guaranteed by public funding [3, 5] in pay-for-performance scheme which was mainly based on the quantity and quality of BPHS, e.g., numbers of patients educated and physical examination forms. Besides, the national public finance for annual BPHS work has increased gradually to 50 Yuan per capita in 2017 [15].

### Rationale for coping

**Fig. 1:**
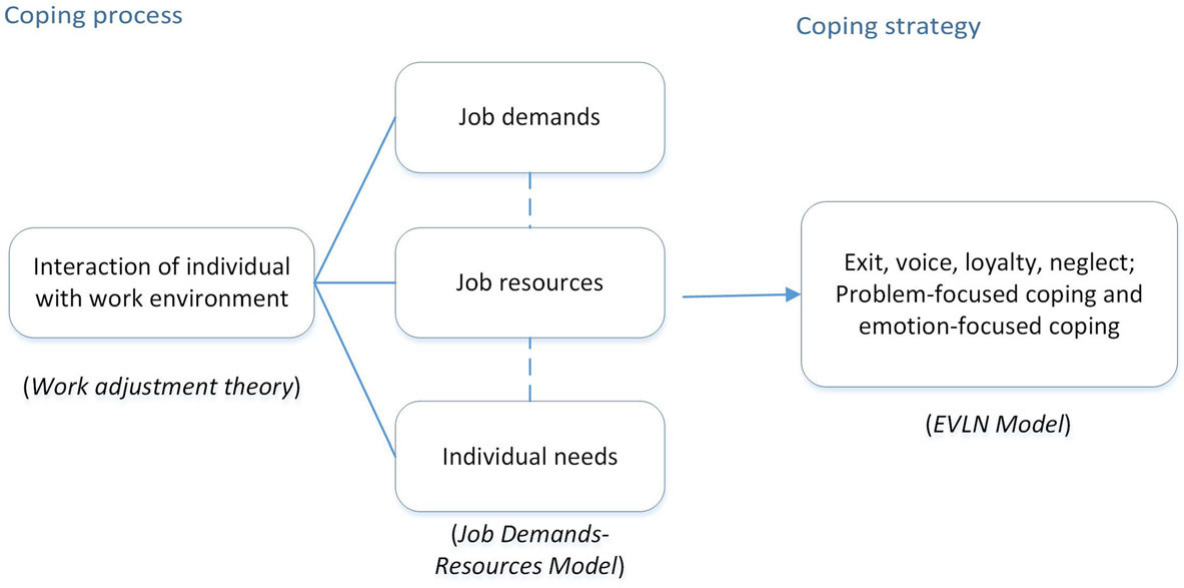
Theoretical framework of primary health workers’ coping with work

The combination of new functions, organization transition, cadres and roles, and public funding has fundamentally changed the landscape of CHS. While these community health reforms have been documented, the question of how PHC providers cope with these processes of successive reform has been largely neglected. Drawing on organizational behavior theories to understand this question (see Fig. 1), such coping can be seen as a process that represents the thoughts and behaviors that people use to manage demands deemed to be stressful [16, 17]. There are generally two approaches of coping: problem-focused coping and emotion-focused coping. Problem-focused coping refers to doing something to alter the source of the stress to prevent or control it whereas emotion-focused coping looks at reducing or managing the emotional distress associated with the situation [17].

The coping of PHC providers with their work is reflected by interaction of the individual with the work environment. The theory of work adjustment [18] indicates that this interaction is to reach a reciprocal correspondence between the work personality and work environment with the individual fulfilling the requirements of the work environment and the work environment fulfilling the requirements of the individual [19]. To achieve such arrangements, coping can be seen as a psychological process of individuals balancing job resources, job demands and individual needs. The extent to which the job meets the needs and values of employees can be referred to as the “job resources” [20] such as income, professional development opportunity, training, and benefit packages. Job requirements from employees for the benefit of the organization can be linked to “job demands” such as workload, work time, work feature, and work skills.

Various coping strategies are embodied in work attitudes and work behaviors as internal and external reactions to the work environment. According to Withey and Michael [21], coping strategies can be categorized into four basic options: exit, voice, loyalty, and neglect, i.e., the EVLN model. If employees are dissatisfied, they can focus on their non-work interests, and “neglect” their unsatisfactory work situation or they can try to improve the situation, through “voice”. Otherwise, they can quit and find a better job (“exit”). For loyalty, they can be motivated and actively support the organization (“active loyalty”); or when they are not so motivated, they can be silent and patient, doing the necessary amount of job as required (“passive loyalty”) [22].

Despite the abundant literature on coping, the potential of coping research in human resource for health has been insufficiently explored. Studies have utilized coping scales to assess the degree of burnout and stress of health workers [23–26], yet very few studies have examined coping strategies related with health reform and work changes. Lemaire and colleagues found some useful coping tools, such as working through stress, talking with co-workers, taking a time out, and using humor [27], yet authors did not translate these into coping strategies. One of the few studies can be found in South Africa where Eyres and colleagues used a qualitative methodology to find that resistance, resilience, and endurance acted as three coping strategies used by health workers in response to health system challenges [28].

The purpose of this qualitative study is to analyze how health reform of CHS led to changing job features for primary health providers. In doing so, it looks to show how PHC providers developed coping strategies to deal with these changes. It provides a valuable contribution to current knowledge gaps regarding health workers’ coping to changes in work contexts across China. Additionally, its findings of health workers’ coping provide valuable learning for other countries undertaking similar health reform efforts.

## Methods

### Design and setting

Our research carried out a range of qualitative interviews with PHC providers about their experiences of coping with successive change in primary care. The study formed one part of the mixed methods project of “the Survey of human resources for Community Health Services” as one part of 2013 National Health Service Survey in China, sponsored by the Ministry of Health.

This national study collected qualitative data from across seven cities from four provincial regions. The purpose of this paper is to present a case study of this material: the experiences of PHC providers in Jinan city. Jinan city was selected as a typical case of CHS development. As the capital city of Shandong province, Jinan is representative of many big cities in China that witnessed significant changes as the result of the market-driven reforms. Jinan has a strong legacy of the planned economy era characterized by many government-owned enterprises and public institutions that had affiliated clinics or hospitals. When CHS was developed, many of these facilities were transformed into a mixed economy of CHS network. As a result, the selection of this case study enables us to capture a range of insights within the context of CHS development highlighted in the previous section.

A semi-structured interview guide was designed to ask respondents about how PHC providers experienced the successive changes surrounding their role and remit. It also looked to gain insights into how these changes influenced their motivation: financial rewards, professional development, relationship, etc.

### Sampling and participants

We used stratified non-probability sampling. First, we used maximum variation sampling to select different ownerships of CHS, e.g., private owner, public hospital, and government-owned public enterprise. Second, we purposively sampled typical CHS of these different ownership structures. Third, in selecting participants, we also applied maximum variation sampling. We used the CHS manager’s help to select PHC providers with rich work experience from every specialty, e.g., public health worker, nurse, specialist doctor, GP, physician of traditional Chinese medicine, and manager of CHS as well.

### Process of investigation

Data collection was carried out by a team from the School of Public Health, Fudan University. The research team received a specific training for this project. Many investigators had interview experience with community health services. They collectively designed and discussed the interview guide and were all familiar with the topic and structure of the interview. In every interview, two interviewers conducted face-to-face interview with one participant. While one interviewer took the role of leading the conversation and exploiting information, the assistant-interviewer aided to supplement questions, taking notes and audio-recording the conversation. Each interview lasted for around 1 h. Discussion was held throughout the research by the research team, and helped form a picture of coping strategies faced by PHC providers. The final interview numbers were based on a saturation point where no new information was being acquired.

### Transcribing and analysis

Data analysis of transcribed interview text used MAXQDA version 2 to manage the data. We applied thematic analysis drawn on Braun and Clarke’s method [29]. Analysis began with the researchers reading and re-reading the transcription and the field notes to get familiar with interviewee accounts of coping. The first stage of analysis employed open coding based on the meaning emerging from the text. Following discussions of these open codes, agreement was reached. In the second stage, these codes were grouped into themes according to their relationships, similarities, and differences. In the third stage, researchers tried to find connections of these themes for making sense of the material. The analysis was subsequently linked to the different theories mentioned in the previous section in order to develop the interpretation of the interview material and deepen our understanding of the PHC providers’ accounts. Data analysis focused on three thematic areas: the arrival of new job characteristics, the coping process, and the strategies used to resolve the tensions arising from the coping process.

Generally, this thematic analysis was in an inductive, bottom-up way [30]. We did not have theoretical presumptions before analysis. However, when the themes were found, we let our data interact with existing theories. Thus, suitable theories were mixed to support understanding the data.

## Results

A total of 12 CHS were investigated. Within these, 30 PHC providers were interviewed (Table 1). Every participant was numbered from Ptp 1 to Ptp 30.

**Table 1:** Numbers of participants by specialty, gender, and CHS owner type

Specialties	Male	Female	Private owner	Public enterprise	Public hospital	Total
Manager of community health services	6	3	0	2	7	9
Public health worker	0	4	1	1	2	4
Nurse	0	7	1	2	4	7
Specialist	1	1	1	0	1	3
General practitioner	1	3	2	0	2	3
Traditional Chinese medicine doctor	2	1	1	0	2	3
Laboratory technician	0	1	0	0	1	1
Total	10	20	6	5	19	30

The following sections present main results of our interviews in Jinan city. They are presented in line with the thematic data analysis that focuses on three thematic areas. First, we introduce the context of changing job characteristics that arose from CHS reform. We then depict the coping process by drawing on the Job Demands-Resources model. Finally, we present how PHC providers adopted different coping strategies drawing on the EVLN model.

### New job characteristics

The balance of two functions of CHS, that is, BPHS and BMS, changed after 2009. On the one hand, basic public health services received increased attention, with an expansion of services and associated guidance from government. On the other hand, basic medical services received less emphasis, with limited service recognition and a lack of guidance regarding what services should be included. In short, public health gained more emphasis than medical care within the functions of CHS.

Our interviews with PHC providers suggested that this change of function balance caused changes in job characteristics. They described three new characteristics of primary health work: less intensive work, trivial work, and heavy workload.

First, the work in CHS was “less intensive” compared with clinical job in hospitals. In hospitals, patients’ conditions were likely to be more complicated and of high risk. Clinical work was interpreted as more stressful whereas in CHS basic public health and basic medical service had a lower risk level. Medical work in CHS became much easier, often referred to as basic transactions such as prescription drug purchases:

Some patients only come to CHS to buy drugs for their long-term NCD, like diabetes (CHS manager, Ptp 1, female).

Work time is much regular compared with hospital work. In hospital, we are faced with patients in suffering, the pressure is overwhelming. In addition, bad physician-patient relationship makes work pressure even greater. In CHS, there is no nightshift, and work is much easier. (One nurse in CHS owned by Public enterprise, Ptp 6, female )

Second, the work in CHS was described as “trivial” compared with that in hospitals. The sense of triviality could be attributed to the BPHS requirement to carry out a range of clerical work related to collecting residents’ health-related information. A nurse (Ptp 12, female) stated:

Work is… also very trivial, tons of trivial things. Every day, I stare at my computer with sore eyes. I have endless forms to fill and materials to dig up, why the public health files can’t be simple? For an eight-month-old baby, I have to complete a development-of-body form, then another form almost the same in the family member file. The same thing happens in quarterly statistical reports, repeated information to be provided. I just don’t see the point.

Third, primary health service was associated with a heavy workload. Although CHS work was much “less intensive” compared with hospital medical work in respect of stress, medical skills, urgency, and risk of health service, the sheer expansion of BPHS tasks still amounted to “heavy” workload. Furthermore, as BPHS tasks expanded, human resource arrangements became relatively inadequate.

After the health reform, workload increased. Tuesday and Thursday were for vaccination. The other days were for community work (note: it means follow-up work and health management in community center or in homes). Computer work (note: that is entering information and filling health profiles) need to be done at the weekend or at night, because workdays were full of other tasks.

… The work was trivial. I, myself, had 3000 residents to follow up and fill their forms. Besides, there’s additional administrative work when supervision and assessment come from “the higher-ups”… These provisional tasks also need our assistance. (A public health worker in public enterprise-owned CHS, Ptp 26, female)

### The coping process

**Fig. 2:**
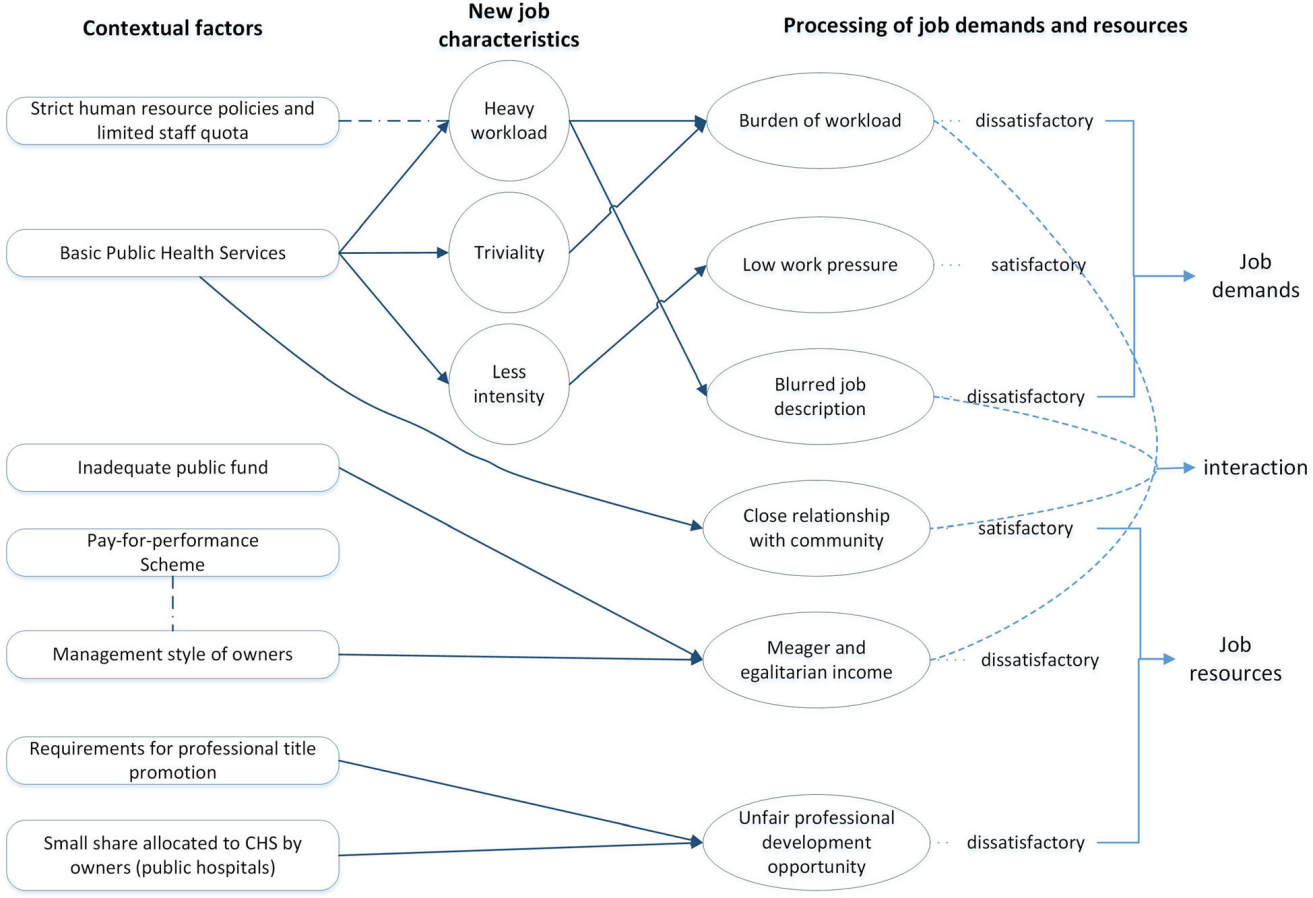
Coping process of primary health care providers in China

The abovementioned new job characteristics together with regulatory contextual factors determined job demands and job resources of community health work. PHC providers went through coping process when they compared job resources and demands with individual needs, and tried to reach consistency between them. We found job resources and job demands were processed by health workers as follows (Fig. 2). The following section details the state of job demands and resources along with the interactions between the two.

#### Job resources

Income, professional development opportunities, and community relationships represented the three major job resources in CHS. Crucially, within the current arrangements, these job resources were not sufficient. We found PHC providers were demotivated by unsatisfactory income and professional development opportunities, but satisfied with close community relationships.

First, the income received a range of complaints. For PHC providers, the income was prescribed by national policy to be somewhere near average level of public institution employees’ [31], regardless of the changing job characteristics and the escalating service package in CHS to meet the needs of the community population. Against this context, most PHC providers felt underappreciated with their meager income being unable to match living standards. One public health worker (Ptp 7, female) commented:

Every day, I work hard with all my heart. Sometimes, stay up all night, only to prepare for an assessment by the government. To my conscience, I can say that I do what my job needs me to do, but the job, the income, definitely does not live up to what I need.

Some PHC providers had lost hope in receiving decent financial rewards.

I don’t believe my income will get a high raise in the future. Because it is not the nation but the Hospital that governs us. Our Hospital thinks it is just OK to have an eventless CHS. They don’t recognize our significance and won’t put much resource in us. (One nurse in government-owned CHS, Ptp 17, female)

They compared their income with their health worker counterparts. One GP (Ptp 11, female) stated:

My salary is about four thousand Yuan a month. That is lower than all my former colleagues in the hospital. Some colleagues get twice my salary. What can I say? Of course I’m not satisfied with my income.

Another cause for complaint about income was egalitarian remuneration plan. In CHS owned by public hospitals or companies, pay-for-performance scheme was hampered by human resource management style from the owner. As a result, traditional remuneration schemes of the owner persisted with salary based on professional title, position, and length of service in the organization rather than the merits and credits of work performance. A manager in one CHS (Ptp 18, male) owned by public enterprise described as follows:

Every half a year, we assess the quantity and quality of service, and the salary should be paid based on the assessment. However, we still follow the Company’s remuneration scheme, which is mainly based on title and position. Employees’ salaries have no notable difference, no matter how good or bad their performance is. Because the Company is our owner, people will say: ‘why we have a different income scheme from our Company?’ The Company has control over us, we can’t carry out the pay-for-performance scheme.

Second, professional development opportunities for PHC providers were perceived as unfair. In 2014, Shandong province, in which Jinan city was located, issued its yearly notification about requirements for promotion to senior medical professional title [32]. In that notification, successful completion of the English test and research publication were required materials for qualification review. The requirements were the same for health workers at a primary level and in hospitals. With promotion rooted in the publication of academic papers and the completion of various assessments, these goals were described as being unrealistic for PHC providers at the grass-root level of health system.

I am 41 now. My professional title promotion was long overdue, but still not realized. 2 years ago, I felt hopeful. Now, I give up hope. You need pass several tests, also research papers and English level certificate. It is no longer about how good my work expertise is. Years go by, my hope ran out. (One specialist in CHS, Ptp 10, male)

There is no favoured nor special policy terms for our (primary health workers’) professional title promotion. We don't have any research project in CHS, my schoolmates working in hospitals have several research projects. We are totally different. People say we primary health workers are like elementary school teachers; they hospital workers are like university teachers. But we had the same requirements for promotion. Isn’t it strange? (One nurse in public hospital owned CHS, Ptp 2, female)

Another cause for unfair promotion opportunity in some CHS founded by public hospitals was that the promotion quota was controlled by hospitals. With limited opportunities for promotion given by these parent hospitals, PHC providers’ motivation for achievement was hindered.

There is very small space for professional rank promotion. It is one big dissatisfaction. Our manager made it really clear that it is impossible to have a vacancy of senior professional title in CHS. It is because we share professional rank quota with the Hospital, it is quite understandable that we primary health workers could only have a small piece. (One GP in CHS, Ptp 11, female)

Third, PHC providers cherished close relationships with the community and valued these as an important job resource. Due to their active and comprehensive work methods in the community, PHC providers had a close relationship with community residents. A doctor of traditional Chinese medicine (Ptp 4, male) shared his pride in these endeavors:

Patients in this community will always come to me after treatment in big hospitals. On my one-hour’s way home, I will be stopped several times by residents and they ask me kinds of questions about their health and life. The community like me. That is what takes me so far till now.

CHS and hospitals have quite different attitudes to patients. In hospital, doctors are too busy to drink water; they will not communicate patiently with residents… But here, we have plenty of time for patients. We actively get in touch with patients. We don’t wait to serve. We try many ways to promote their health, and we explain carefully what their conditions are. Finally, we build very good relationship with the community. (One doctor of Traditional Chinese Medicine, Ptp 13, male)

#### Job demands

Our interviews found that job demands were affected by the new job characteristics emerging from BPHS. PHC providers welcomed the low work pressure of the CHS, yet they complained that the work became trivial, monotonous, and heavy. Blurred job description was another unsatisfactory job demand.

First, when describing work pressures, those who once worked as hospital medical workers appreciated the “less intensive” job of CHS. One nurse (Ptp 2, female) told us:

I was once in the operation room of a hospital, and had to work night shift back then. Unfortunately, my health got worse. I developed Ventricular Ectopic Beat (a heart disease), so I asked to be transferred here in CHS. Now the work is much less intensive. For that, I think it is a good job.

Second, the heavy workload associated with the repetitive “trivial” BPHS work was deemed a burden by PHC providers. One manager of CHS (Ptp 1, female) revealed the burden from the magnitude of BPHS work:

Too many paperwork, and too many follow-up tasks! We need one or two full-time employees to do the daily, weekly, monthly and yearly reports. But, we can’t afford that extra hands, everyone is doing two men’s job.

In CHS … tasks are diverse, trivial and overloaded. Honestly, the stress caused by BPHS is no less. (One nurse in CHS owned by Public enterprise, Ptp 6, female )

Third, job description blurred. New primary health care model requires BPHS to be provided in a GP team, which to some extent caused role transformation of PHC providers. However, a more important cause for blurred job description lies in policy factors. Hampered by bureaucratic procedures and limited public finance, recruitment of additional staff proved to be a challenge. A national policy in 2006 stipulated staff quota calculation method of CHS based on community population [33], which remained valid after 2009 when primary health work tremendously expanded. Consequently, with a heavy workload and lack of human resource, many PHC providers had to undertake extra responsibilities beyond their job description. Roles and responsibilities were often blurred causing role conflicts. A GP in a CHS (Ptp 9, female) said:

After the BPHS was carried out, the workload almost tripled. But the staff number stay the same. As a GP, my position is not fixed. I do both medical and public health works, and whatever work that is short of hands. Sometimes I became a social worker, visiting people and trying to solve their problems that may not directly relate to their health. I don’t know what my position is.

#### Interaction between job demands and job resources

The interaction between job resources and job demands created a range of experiences for health workers in CHS. First, an “aggravation effect” was frequently recognized as heavy BPHS work and relatively low reimbursement intertwined. A public health worker in CHS (Ptp 7, female) stated:

The BPHS work is tremendous. If there is enough reimbursement for human resource cost things will be different. But the fund is limited, only adequate for consumable materials. Many PHC providers do tasks beyond their own job, with no extra pay. It really harms our motivation.

Second, an “alleviation effect” took place where close relationships with community and blurred job description facilitated day-to-day delivery. A considerable number of PHC providers were former specialists in primary level clinics or hospitals. For them, the mixed role of a doctor and clerk and social worker, rather than being a clinician first and foremost, was hard to accept. Nevertheless, their experience of working in the community resulting in close community relationships provided affectional reward. This reward gradually persuaded CHS doctors to be PHC providers rather than hospital specialists. These close relationships with community alleviated unpleasant experiences of mixed role of BPHS and BMS work. One GP (Ptp 3, female) was a former specialist, and she admitted her ambivalence about CHS work:

In CHS, I do many things that are not medical work. I feel it is difficult to improve my clinical skill level. Without a high level of clinical skill, patients won’t respect you. They know our skill isn’t as good as doctors in hospitals… Anyway, they like our service, because we talk with them, we are kind and caring.

Residents love to come here for health service. They know us well, like old friends. But when they get some ‘real problems’, they prefer going to hospitals. They say ‘the community hospital (note: that is how residents and many PHC providers call CHS) is just doing administrative work.’ We honestly are not as good as hospital clinicians. (One public health worker in CHS owned by public hospital, Ptp 14, female)

### Coping strategies

The gap between job demands and job resources meant that for most PHC providers dissatisfaction and low motivation became a prevailing work attitude. As a result, our interviews found that PHC providers used the coping strategies of exit, passive loyalty, and compromise to resolve the situation.

First, after weighing up job resources against job demands, some PHC providers chose to exit. Dissatisfaction was particularly pronounced among younger, more ambitious workers, affected by unsatisfactory income and promotion opportunities. Dissatisfaction forced them to exit the job:

For myself, I don’t care so much. But those ambitious ones, eager to climb up the ladder, have something else in mind. They won’t stay here for long, the income is too low for them. Young people have responsibilities, to support family, to buy house and car, the salary is not enough for these needs. They will jump to hospitals, or try to be civil servants or change to another career. (A TCM doctor in public-hospital-owned CHS, Ptp 30, female)

Second, there was “passive loyalty”. Despite their dissatisfaction with the job, some PHC providers could not find a way to change the work conditions. Their dissatisfaction was not strong enough for them to exit. They continue in their job with minimal effort. Passive loyalty was mostly towards public health work and more prevalent among older workers.

The income is low, and no favourable policies to motivate us. We have to do whatever the work demands. When patients visit, some fellow workers serve them well. But when it comes to going out to the community and follow-up, they are unwilling and find the reason not to go out. (One nurse in government-owned CHS, Ptp 17, female)

Third, some adjusted their motivation level by lowering expectations. They compromised and chose to focus on the positives to buffer dissatisfaction, which could bring about a new correspondence between the individual and the work.

I will do what I am required to do. I just want to live quietly. Professional title will be elevated when the time comes. I will not leave this organization, I get used to the job. Besides, I don’t think I can find a better job. Of course there is pressure, but not too much. Though there is a heavy workload, the atmosphere is harmonious, so we feel happy. (A public health worker in public enterprise-owned CHS, Ptp 26, female)

These compromised PHC providers started to distinguish themselves from hospital medical workers and were more committed to primary health care. Their interpretation of job orientation was re-constructed.

I prefer community health work to hospital medical services. Here in CHS, we have systematic preventive services, which cannot be provided by hospital … We build trust through medical services and get to know the patient’s lifestyle. …Yes, we can’t provide better drugs, but we can help you control your blood sugar, which the hospitals are not able to do. (A public health worker in public-hospital-owned CHS,Ptp 19, female)

## Discussion

Against the backdrop of CHS reform from 2009, PHC providers represent a considerably new healthcare profession in China. Within the reform arrangements, BPHS have become a major service function in CHS. Our findings show how these changes have led to a range of new job characteristics associated with trivial work, heavy workload, and less intensity. These changing job characteristics, together with contextual factors, have influenced PHC providers’ processing of job demands and job resources. In processing job resources and job demands, there demonstrated two ways of interaction: aggravation and alleviation. The processing and interaction of job demands and resources have led to a range of coping strategies in the form of exit, passive loyalty, and compromise.

Our findings provide much needed qualitative perspectives regarding the current situation for PHC providers. The findings support the range of quantitative studies in China showing how PHC providers’ satisfaction with income and professional development were lower than other facets of job satisfaction [34–36]. PHC providers have quit their job because of low income and limited opportunities for professional development [37]. Public health workers tend to be more dissatisfied with workload than physicians and nurses [35].

Policies are powerful contextual factors, which can influence job characteristics and further trigger a series of coping process of health workers. In China, the dissatisfaction with professional development and heavier workload can be partly attributed to obsolete personnel regulations. As mentioned in results, policy of staff quota remained the same when the work of primary health care underwent massive changes; incoming level prescribed by policy was rigid and lagging behind job demands; English test was made a must for professional title promotion when it is not a necessary skill for most PHC providers [31–33].

International studies also showed how policy change in primary care had a powerful influence on job characteristics, health workers’ attitudes, and their coping strategies. Health policy initiatives in the 1990s in Ontario had been intended to enhance community health care just like in China. Similarly, workload increased, especially clerical work [38]. Research in Tanzania found expanding workload and limited human resources resulted in additional duties beyond job description, and demotivated PHC providers [39]. Health workers’ coping strategies are often triggered by ineffective policies. Comparable but much worse than in China, performance problems of health workers in public health sectors in Ethiopia, including absenteeism, shirking, and pilfering drugs, were understood as normal methods to deal with inadequate compensation, a lack of necessary supplies, and a command-and-control mode of government regulations that were perceived as unfair and lacking in transparency [40].

Coping strategies are somehow contingent on specific work contexts. If we turn to our use of the EVLN model, our research did not find “voice” or “neglect”, but did discover a coping strategy something akin to “compromise”. Our findings support such a view that compromise can be seen as a positive coping strategy, adaptive to the changing environment and finding ways to fit into the new primary health care model. While research often assumes emotion-focused coping as a passive approach [24], the PHC providers in our study used compromise to facilitate a re-orientation of job role, including a positive cognitive restructuring to challenging situations [41]. The PHC providers’ experience supports findings from other studies showing how emotion-focused coping can lead to changes of aspirations and goals in order to reduce gaps between perceived requirements and resources [42]. Through compromise, one can achieve satisfaction and focus on current work actively [43].

Quantitative arm of this project on person-organization fit found that needs of relatedness were basically satisfied, including co-worker relationship, resident respect, and work security, while achievement needs such as career development, income, and learning opportunity were obviously not satisfied [44]. It is probably due to different experience of achievement needs and relatedness needs that PHC providers compromised their achievement needs and resorted to relatedness needs to reach inner peace. That also explains why another paper from a quantitative arm of this project found that PHC provider health workers were basically satisfied with the work and generally willing to stay in CHS [45]. PHC providers chose a strategy of compromise and adapted themselves to work in CHS.

There are three possible reasons for which “voice” as a coping strategy was not found in this study. First, as all PHC providers work in teams of five to seven colleagues, the equity in workload made the need to exercise voice diminish [16]. Second, within the context of China, voice is not an effective coping strategy. Health reform policies in China seldom consider workers’ opinions, and consequent job changes are often beyond what PHC providers can improve [46–48]. Third, when excessive changes were brought into the work, negative coping rather than problem-focused coping is more likely to develop, including resistance, withdrawal, and expressed cynicism [49].

Passive loyalty could also be useful for future adaptive coping. One study of South African physicians coping with a defective health system found endurance, resistance, and resilience as ways of coping [28]. Passive loyalty could be seen as a midpoint of resistance and endurance. While it reduces the effort to resist and protect one’ interests and values, it nonetheless keeps work going on. In this way, passive loyalty are not merely passivity but rather allowing future adaptive coping, e.g., exit or compromise.

### Policy and research implications

The policy implications of our findings suggests that there is a role for central and local governments to foster positive coping through further investment in necessary resources for CHS (e.g., financial input) and redesigning inefficient human resource policies. Another practicable method is through empowering health workers in decision-making, review, and adaption of health reform policies, to make “voice” a possible and useful coping method. More work is needed to support the transformation of PHC providers’ job role from hospital medical work to primary health work, e.g., training courses for job role orientation.

For future research, it is advisable to carry out a follow-up qualitative and quantitative research and examine evolution of coping strategies in light of any further changes being introduced. Another suggestion would be further exploration of work-life balance, and how being a healthcare provider interacts with other aspects of Chinese society. Given the fact that current CHS reform efforts are commonly associated with coping of health workers, a service improvement research agenda is required to assess different ways in which policy can be improved.

### Limitations

There are two limitations to this study. First, the interviewees were prone to drawbacks of the reform policies and complaint about the job, while improvement of CHS work condition was largely neglected in the interview. This could be because excessive changes can amplify cynicism and complaint in health workers [49]. Second, researchers’ mindset may be restricted by the established interview structure that mostly focused on work motivation and work attitudes. Work-life balance and work-family conflicts were not explored in the study.

## Conclusions

The reform of community health services in China has had a range of consequences for primary health care providers. Our research has found that since 2009, community health services have been perceived as “less intensive” than hospital medical work, associated with trivial and heavy tasks. Heavy workloads in tandem with limited staff quota have given rise to blurred job description. Unsatisfactory job resources such as low income and unfair professional development opportunities have demotivated PHC providers, especially those with strong achievement needs. That said, the comprehensive work associated with BPHS across the community has resulted in close relationships with community residents. As a result, the combined effect of job demands and job resources has led PHC providers to develop three coping strategies. Some PHC providers decided to exit, some demonstrated passive loyalty, and others opted to adjust their expectations and compromise with the new model of primary health care. Our study provides a valuable contribution to context-related coping research in the area of human resources for health.

## Abbreviations

BMS: Basic medical services, a loose range of basic medical care provided by CHS with unstable government fund. BMS and BPHS comprise the main function of CHS.
BPHS: Basic public health services, a government-funded package of basic public health care provided by CHS.
CHS: Community health services, the facilities that provide primary health care and family medicine in China.
EVLN model: Exit-Voice-Loyalty-Neglect model, coping strategies can be categorized into four basic options: exit, voice, loyalty, and neglect.
GP: General practitioner, a family doctor who mostly work in CHS.
PHC providers: Primary health care providers, those who work in CHS, including cadres of general practitioners, specialists, nurses and public health workers, as well as medical lab technician and doctors of traditional Chinese medicine.
TCM: Traditional Chinese medicine

## References

[1] The Central Committee of CPC, the State Council of PRC: The Decision on Health Reform and Development. Beijing: The State Council of PRC; 1997.

[2] Li H, Yu W (2011). Enhancing community system in China's recent health reform: an effort to improve equity in essential health care. Health Policy.

[3] Yip W, Hsiao W (2009). China's health care reform: a tentative assessment. China Econ Rev.

[4] Xiao N, Long Q, Tang X, Tang S (2014). A community-based approach to non-communicable chronic disease management within a context of advancing universal health coverage in China: progress and challenges. BMC Public Health.

[5] Liu Q, Wang B, Kong Y, Cheng KK (2011). China's primary health-care reform. Lancet.

[6] General Office of the State Council of PRC (2015). The National Health Service System Plan (2015-2020).

[7] The State Council of PRC (2011). A guide for establishing the system of general practitioners.

[8] Bhattacharyya O, Yin D, Wong ST, Chen B (2011). Evolution of primary care in China 1997-2009. Health Policy.

[9] Chen Z (2009). Launch of the health-care reform plan in China. Lancet.

[10] Yip W, Hsiao WC, Chen W, Hu S, Ma J, Maynard A (2012). Early appraisal of China's huge and complex health-care reforms. Lancet.

[11] Ministry of Health of PRC (2009). National Guideline for basic public health services 2009.

[12] National Health and Family Planning Commission of PRC (2017). National Guideline for basic public health services 2017.

[13] Zhao Y, Chen R, Wang B, Wu T, Huang Y, Guo A. General practice on-the-job training in Chinese urban community: a qualitative study on needs and challenges. PLoS One. 2014;9 10.1371/journal.pone.0094301 PMC3984120 24728399

[14] Zhao Y, Cui S, Yang J, Wang W, Guo A, Liu Y, Liang W (2011). Basic public health services delivered in an urban community: a qualitative study. Public Health.

[15] General Office of State Council of PRC: Notification about key tasks of deepening the health care reform in 2017. Beijing: National Health and Family Planning Commission online; 2017. [http://www.gov.cn/zhengce/content/2017-05/05/content_5191213.htm].

[16] Astvik W, Melin M (2013). Coping with the imbalance between job demands and resources: a study of different coping patterns and implications for health and quality in human service work. J Soc Work.

[17] Lazarus RS, Folkman S. Stress, appraisal, and coping. New York: Springer Pub. Co.; 1984.

[18] Dawis RV, Lofquist LH (1976). Personality style and the process of work adjustment. J Couns Psychol.

[19] Rounds JB, Dawis RV, Lofquist LH (1987). Measurement of person environment fit and prediction of satisfaction in the theory of work adjustment. J Vocat Behav.

[20] Bakker AB, Demerouti E (2007). The job demands-resources model: state of the art. J Manag Psychol.

[21] Withey MJ, Cooper WH (1989). Predicting exit, voice, loyalty, and neglect. Adm Sci Q.

[22] Rusbult CE, Farrell D, Rogers G, Mainous AG (1988). Impact of exchange variables on exit, voice, loyalty, and neglect: an integrative model of responses to declining job satisfaction. Acad Manag J.

[23] Guveli H, Anuk D, Oflaz S, Guveli ME, Yildirim NK, Ozkan M, Ozkan S (2015). Oncology staff: burnout, job satisfaction and coping with stress. Psycho-Oncology.

[24] Howlett M, Doody K, Murray J, LeBlanc-Duchin D, Fraser J, Atkinson PR (2015). Burnout in emergency department healthcare professionals is associated with coping style: a cross-sectional survey. Emerg Med J.

[25] Koh MYH, Chong PH, Neo PSH, Ong YJ, Yong WC, Ong WY, Shen MLJ, Hum AYM (2015). Burnout, psychological morbidity and use of coping mechanisms among palliative care practitioners: a multi-centre cross-sectional study. Palliat Med.

[26] Schreuder JAH, Plat N, Mageroy N, Moen BE, van der Klink JJL, Groothoff JW, Roelen CAM (2011). Self-rated coping styles and registered sickness absence among nurses working in hospital care: a prospective 1-year cohort study. Int J Nurs Stud.

[27] Lemaire JB, Wallace JE. Not all coping strategies are created equal: a mixed methods study exploring physicians' self reported coping strategies. BMC Health Serv Res. 2010;10 10.1186/1472-6963-10-208 PMC2914035 20630091

[28] Eyles J, Harris B, Fried J, Govender V, Munyewende P. Endurance, resistance and resilience in the south African health care system: case studies to demonstrate mechanisms of coping within a constrained system. BMC Health Serv Res. 2015;15 10.1186/s12913-015-1112-9 PMC4588259 26420405

[29] Braun V, Clarke V (2006). Using thematic analysis in psychology. Qual Res Psychol.

[30] Boyatzis RE (1998). Transforming qualitative information: thematic analysis and code development.

[31] Ministry of Human Resources and Social Security of PRC: A guideline about implementing pay-for-performance in public health and primary health institutions. Beijing; 2009. [http://rsj.shangluo.gov.cn/shetao/201404/20140424173241_28.htm]. Accessed 21 May 2017.

[32] Health and Family Planning Commission of Shandong Province (2014). A notification about materials required for qualification review of senior medical professional title.

[33] Central Quota Office of PRC (2006). A guideline of institution configuration and quota standard for urban community health services.

[34] Ding H, Sun X, Chang W-W, Zhang L, Xu X-P. A comparison of job satisfaction of community health workers before and after local comprehensive medical care reform: a typical field investigation in Central China. PLoS One. 2013;8 10.1371/journal.pone.0073438 PMC3772794 24058472

[35] Shi L, Song K, Rane S, Sun X, Li H, Meng Q (2014). Factors associated with job satisfaction by Chinese primary care providers. Prim Health Care Res Dev.

[36] Luo ZN, Bai X, Min R, Tang CM, Fang PQ. Factors influencing the work passion of Chinese community health service workers: an investigation in five provinces. BMC Fam Pract. 2014;15 10.1186/1471-2296-15-77 PMC4012170 24885642

[37] Meng QY, Yuan J, Jing LM, Zhang JH. Mobility of primary health care workers in China. Hum Resour Health. 2009;7 10.1186/1478-4491-7-24 PMC2661043 19292911

[38] Cohen M, Ferrier B, Woodward CA, Brown J (2001). Health care system reform - Ontario family physicians' reactions. Can Fam Physician.

[39] Mbilinyi D, Daniel ML, Lie GT. Health worker motivation in the context of HIV care and treatment challenges in Mbeya region, Tanzania: a qualitative study. BMC Health Serv Res. 2011;11 10.1186/1472-6963-11-266 PMC3214153 21992700

[40] Lindelow M, Serneels P (2006). The performance of health workers in Ethiopia: results from qualitative research. Soc Sci Med.

[41] Collins S (2008). Statutory social workers: stress, job satisfaction, coping, social support and individual differences. Br J Soc Work.

[42] Hertel G, Rauschenbach C, Thielgen MM, Krumm S (2015). Are older workers more active copers? Longitudinal effects of age-contingent coping on strain at work. J Organ Behav.

[43] Büssing A, Bissels T (1998). Different forms of work satisfaction: concept and qualitative research. Eur Psychol.

[44] Zhang M, Wang W, Wang Y, Qian Y, Wang Y, Xu L, Yan F. Analysis on the person-organization fit for community health workers in China. Chinese Journal of Health Policy. 2016:27–32.

[45] Zhang M, Yan F, Wang W, Li G. Is the effect of person-organisation fit on turnover intention mediated by job satisfaction? A survey of community health workers in China. BMJ Open. 2017;7 10.1136/bmjopen-2016-013872 PMC5337699 28399513

[46] Beijing Commission of Chinese Peasants and Workers Democratic Party: The health care reform should listen to medical staffs' voice. Beijing: Bejing Observation; 2015. p. 20–21.

[47] Ning F. Health reform: could you hear the voice of the frontline health workers? Finance Sina website; 2014. [http://finance.sina.com.cn/zl/china/20140315/100918517460.shtml]. Accessed 21 May 2017.

[48] Yixuejie. Success is not possible for health reform at present.Yaozui net; 2017. [http://www.yaozui.com/p/135918]. Accessed 11 July 2017.

[49] Johnson KJ, Bareil C, Giraud L, Autissier D (2016). Excessive change and coping in the working population. J Manag Psychol.

